# Productivity in relation to organization of a surgical department: a retrospective observational study

**DOI:** 10.1186/s12893-022-01563-6

**Published:** 2022-03-24

**Authors:** Johan Eriksson, Philip Fowler, Micael Appelblad, Lena Lindholm, Malin Sund

**Affiliations:** 1 Department of Surgical and Perioperative Sciences, Umeå University, 901 87 Umeå, Sweden; 2 Department of Nursing, Umeå University, 901 87 Umeå, Sweden; 3 Department of Statistics, Uppsala University, 751 20 Uppsala, Sweden; 4 Department of Public Health and Clinical Medicine, Umeå University, 901 87 Umeå, Sweden; 5 Department of Surgery, University of Helsinki, 000 14 Helsinki, Finland

**Keywords:** Operating room, Perioperative management, Logistics

## Abstract

**Background:**

Responsible and efficient resource utilization are important factors in healthcare. The aim of this study was to investigate how total case time differs between two differently organized surgical departments.

**Methods:**

This is a retrospective observational study of a cohort of patients undergoing elective surgery for breast cancer or malignant melanoma in a university hospital setting in Sweden. All patients were operated on by the same set of surgeons but in two different surgical departments: a general surgery (GS) and a cardiothoracic (CT) surgery department. Patients were selected to the two departments from a waiting list in the order of referral for surgery. The effect of being operated on at the CT department compared to the GS department was estimated by linear regression.

**Results:**

The final study cohort comprised 349 patients in the GS department and 177 patients in the CT department. Both groups were similar regarding surgical procedures, American Society of Anesthesiologists’ score, body mass index, age, sex, and the skill level of the operating surgeon. These covariates were included in the linear regression model. The total case time, defined by the Procedural Time Glossary as room set-up start to room clean-up finish, was significantly shorter for the patients who underwent a surgical procedure at the CT department compared to the GS department, even after adjusting for the background characteristics of the patients and surgeon. After adjusting for the selected covariates, the average difference in total case time between the two departments was − 30.67 min (p = 0.001).

**Conclusions:**

A significantly shorter total case time was measured for operations in the CT department. Plausible explanations may be more beneficial organizational factors, such as staffing ratio, skill mix in the operating room team, and working behavioral aspects regarding resource utilization.

**Supplementary Information:**

The online version contains supplementary material available at 10.1186/s12893-022-01563-6.

## Background

The Swedish healthcare system has experienced a steady increase in costs over the last few years. Between the years 2012 and 2015, this increase was estimated to be approximately 36 billion Swedish Krona, amounting for an approximate increase of 8.4% during this time-period. Costs for inpatient care increased by 5.8% during this time-period and comprised 20.6% of the total budget for the Swedish healthcare system [1]. Surgical interventions in an inpatient setting have increased 35.5% over the last two decades [2] and, between the years 2005 and 2015, the proportion of outpatient surgical interventions rose from 57.7 to 72.6% [3], leading to an overall expansion of surgical activity [2].

This increase in demand for surgical care puts a strain on the health care system in terms of both the economy and working environment, which can threaten the quality of surgical care. In this setting, there are incentives to streamline organizational parts of the system in attempts to produce more health care for the same monetary investment. An operating room (OR) in the United Kingdom has been calculated to costs about £12–£20 per minute of surgical activity [4]. Corresponding numbers for the United States are $22–$133 per minute, with an average of approximately $62 [5], making efficiency in the operation room a high priority.

Studies have found that working in a parallel fashion (e.g., using induction rooms) is more efficient than working sequentially [6–8]. Standardization of surgical practice is also a cornerstone in reducing wasted time [9] and, though every patient is unique, the different key phases in the operation room are consistent enough for any given surgical procedure are consistent enough to be classified into operative and non-operative time. This enables the team to improve its efficiency by outlining and streamlining these phases [10]. Previous studies have tried to establish organizational factors for improving OR turnover time (TOT) [11–13] or different organizational interventions, such as lean and six sigma [14, 15]. However, we cannot find studies that include a constant group of surgeons that ambulate between and operate within two different types of long-term established ORs.

The aim of this study was to investigate how total case time, defined by the Procedural Time Glossary (PTG) [16] as room set-up start (RSS) to room clean-up finished (RCF), differs between two differently organized surgical departments when the same set of surgeons perform the same type of surgeries. Our hypothesis is that organizational differences between the two departments could contribute to any differences in outcome between them.

## Methods

### Data source and collection

The operation planning and scheduling software Orbit (version 4.25.4, EVRY Healthcare System AB, Sweden) was used to extract perioperative patient records for patients with primary diagnoses corresponding to either elective surgery for breast cancer or malignant melanoma at Umeå University Hospital in Sweden between the years 2012 and 2016. We chose the time interval to maintain a consistent group of surgeons and personnel at each department. To prevent bias, data from the Orbit software from late 2016 were not included, as there had been a major update to the software with a completely new user interface, which may have affected the registration process. Orbit contains both patient- and procedure-specific information gathered according to a predetermined protocol, though with some freedom for comments and notes; therefore, it offers a good basis for data collection.

### Study setting and selection of study groups

Patients undergoing surgical intervention for breast cancer or malignant melanoma were given surgery time slots at one of two surgical departments, namely at a general surgery (GS) department or a cardiothoracic surgery (CT) department. We provide an organizational comparison of these two departments in Table 1.

**Table 1:** Comparison of organizational factors between the two analyzed departments

Organizational factor	GS Department	CT Department
Dedicated anesthesiologist	Shared resource (one responsible for multiple ORs)	Allocated to one OR
Access to auxiliary personnel outside of the OR	Sometimes	Always
Operating room nurses both scrubbed in and in assisting/circulating role	Never	In 77.4% of operations
Lunch breaks for OR team	Staff usually replaced during lunch	Team-member interchange
Same working hours for the whole team	Usually	Always
Total number of personnel at each department	~ 80	~ 40
Maximum number of personnel in the OR during surgery	6	6
Working hours	07:00–16:30	07:00–16:00

*GS* general surgery, *CT* cardiothoracic surgery, *OR* operating room

Personnel scheduling operations allocated the patients from a surgical waiting list with the aim of maximizing OR utilization. Thus, the selection to each department thus mainly depended on when the patient was referred for surgery, typically being allocated in the order in which they were referred, as well as the expected duration of the operation (shorter surgeries may fit into shorter time slots in the schedule).

The study design was chosen based on the assumption that the department to which a patient was assigned was unrelated to patient characteristics (i.e., that the assignment in practice could be considered randomized). However, after 2013, more available resources were prioritized for breast cancer surgery, which affected the referral of patients with malignant melanoma, decreasing the number of melanoma patients referred to the CT department. In addition, the CT department was utilized only 1–2 days/week for the included surgery, whereas the included procedures were, to some extent, performed 5 days/week at the GS department. For more information about the allocation process, see Additional file 1: Fig. S1. In order to keep the data set manageable, the time frame for the GS department was reduced to only allow inclusion between January and May of each year, whereas patients from the CT department (with a smaller patient base) were included during the whole study period. Therefore, the overall conclusions of the study were not altered by allowing the estimated effect to vary between procedure groups.

### Inclusion/exclusion criteria and data on subgroups

To be eligible for inclusion in the study, a patient had to be admitted for elective surgery under general anesthesia for either breast cancer or malignant melanoma. The surgical procedure was to be performed Monday to Thursday, excluding bank holidays, between January and May at the GS department or January to December at the CT department. Three criteria were used to classify the different procedure groups: (1) according to diagnostic and intervention codes, as classified by the ICD-10 standard [17], (2) the type of procedure they underwent, and (3) the Classification of Care Measures (i.e., CCM codes). The CCM codes include codes for surgical procedures used in Sweden, a classification generated by the Swedish National Board of Health and Welfare [18]. The procedures were divided into more extensive (Group 1) and limited (Group 2) procedures for breast cancer and operations for malignant melanoma (Group 3). For more information on the procedure code-classification, see Table 2. Note that, for each patient, the ICD-10 classification was only the primary classification in each of the procedure groups; that is, certain specific interventions, such as lymph node biopsy (PJA10) or reconstruction using tissue flap and suture (ZZR70), can be performed in each case without affecting which procedure group includes the patient. Any patients having diagnoses/interventions not listed in Table 2 were excluded from the analysis. Patients undergoing autologous or implant-based immediate breast reconstruction, cases lacking registered values for body mass index (BMI), and those with time registration errors were also excluded.

**Table 2:** Included diagnosis and intervention codes for each procedural group

Procedure group 1	Procedure group 2	Procedure group 3
Diagnosis code breast cancer (C50.*)	Diagnosis code breast cancer (C50.*)	Diagnosis code malignant melanoma/unspecified malignant tumor of the skin (C43.*–C44.*)
Modified radical mastectomy (HAC22)	Resection of breast tissue ± lymph node biopsy ± reconstruction using tissue flap and suture (HAB** ± PJA10 ± ZZR70)	Lymph node clearance (PJD**)
Resection of breast tissue + axillary lymph node dissection (HAB** + PJD42)	Simple mastectomy ± lymph node biopsy (HAC** ± PJA10)	Wide local excision ± lymph node biopsy (QAE** QBE**, QCE**, QDE** ± PJA10)
Axillary lymph node dissection (PJD42)	Local excision of breast tissue (HAF**)	
	Biopsy and incision of breast tissue (HAA10)	
	Other minor correction of breast tissue (HAD99)	
	Re-operation after previous breast surgery (HWA00, HWC00, HWE00, HWD00, HWW99)	

^*^Each sub-code available to the main code is included, e.g., C50.* includes every code between C50.1 to C50.9

^**^The same principle applies to intervention codes. When determining which procedure group a specific case should be included in, one must combine at least two of the listed codes. For example, a patient with C50.1 and PJD42 is sorted into procedure group 1, whereas one with C50.1 and HAB20 ± PJA 10 is sorted into procedure group 2

### Basic characteristics of the two departments

As listed in Table 1, the anesthesiologists at the GS department handle 2–3 ORs, whereas they are only responsible for one OR at the CT department. At the GS department, there is often a lack of personnel outside the OR assisting with transportation of pathology samples and equipment set-up. The GS department uses a team of one operating room nurse (ORN) and one enrolled nurse during the surgical procedure, whereas the CT department strives to always have two ORNs. During lunch breaks, the GS department usually switches the whole OR team, whereas the CT department has their auxiliary personnel switched in one member at a time. At the GS department, staff can start working at different hours (e.g., scrub nurse starts at 07:00 whereas the circulating nurse starts at 07:30), whereas the staff in the CT department always start at the same hour. There are also some differences in the total number of personnel at each department, whit the GS department having a staff of approximately 80 and the CT department having a staff of approximately 40. Both departments maintain the availability of resources for any emergency surgical procedures.

### Statistical analysis

The outcome variable of interest was the total case time in minutes. This is defined as the time elapsed from when the OR team starts case preparation until the room is cleared and ready for the next operation. The difference in the average total case time between patients undergoing surgery at the CT and GS departments was the primary end point.

Several covariates were available, such as clinical, staffing and patient characteristics (Table 3). Only covariates measured before assignment to the specific department were included in the analysis, as adjustment for covariates determined after the assignment can introduce bias in the estimated effect of interest [19].

**Table 3:** Summary of the characteristics and units of measurement

Covariate	Description
Age	Patient age (years)
BMI	Body mass index (kg/m^2^)
ASA score	American Society of Anesthesiologists’ physical status classification (ASA 1–4)
Patient sex	Male or female
Total number of personnel^a^	The total number of personnel present any time during of the operation (n)
Surgical trainee present	If a surgical trainee or surgical assistant is present during the operation (Yes/No)
Total number of students^a^	The total number of students present any time during of the operation (n)
Medical/nursing student present^a^	If a medical or nursing student is present during the operation (Yes/No)
Procedure group 1	If the patient is undergoing an extensive surgical procedure for breast cancer (Yes/No)
Procedure group 2	If the patient is undergoing a limited surgical procedure for breast cancer (Yes/No)
Procedure group 3	If the patient is undergoing a surgical procedure for malignant melanoma (Yes/No)
Consultant present	Main surgeon is a consultant (Yes/No)
High-volume surgeon present	If the main surgeon performs > 50 procedures/year (Yes/No)

^a^Covariates not used in the analysis

To increase the precision in the estimated effect of the department allocation, and to reduce bias because of differences in covariates between the two departments, we adjusted for covariates of clinical importance (Table 3). Missing values were handled through listwise deletion (i.e., the removal of observations with any missingness). A linear regression model was used to estimate the average difference in total case time between the two departments. In addition to patient-related covariates (patient age, BMI, sex, ASA score), the procedure group and three additional covariates were included in the regression model: the presence of a surgical trainee/assistant surgeon, whether the surgeon was a consultant, and the surgeon’s level of experience. These were pre-treatment variables because the staff schedules were set in advance, before allocation to the departments. In terms of the choice of patient related covariates, we argue that ASA score provides a rather well-summarized picture of the severity of the patient’s condition [20]. According to Luedi et al. [21], estimates of case duration can be improved by including patient age. BMI is a significant, albeit weak, predictor of difficulties with tracheal intubation and the prolongation of non-operative time [22]. Unfortunately, there were some missing values for this covariate. Although listwise deletion leads to unbiased estimates of the average treatment effect if the values are missing completely at random [23], additional efficiency can be gained in randomized studies by imputing the missing data. We investigated the sensitivity of our results to deviations from the missing completely at random assumption by systematically imputing higher BMI values at one department than at the other. If the data are missing completely at random for a covariate in a randomized study, then imputing the sample mean into the regression model for the missing data and adding an indicator variable for missingness typically leads to estimates with lower standard errors [24]. The results were largely unchanged by this imputation scheme. We argue that, even if the missing BMI values were systematically higher at one of the two departments, our qualitative conclusions are the same, at least if the missing BMI values are similar in magnitude to those observed. Therefore, we opted to include all these covariates in our model.

We carried out all statistical analyses in R (software version 3.5.1, R Foundation for Statistical Computing, Vienna, Austria, 2018) [25].

## Results

### Study group

**Fig. 1:**
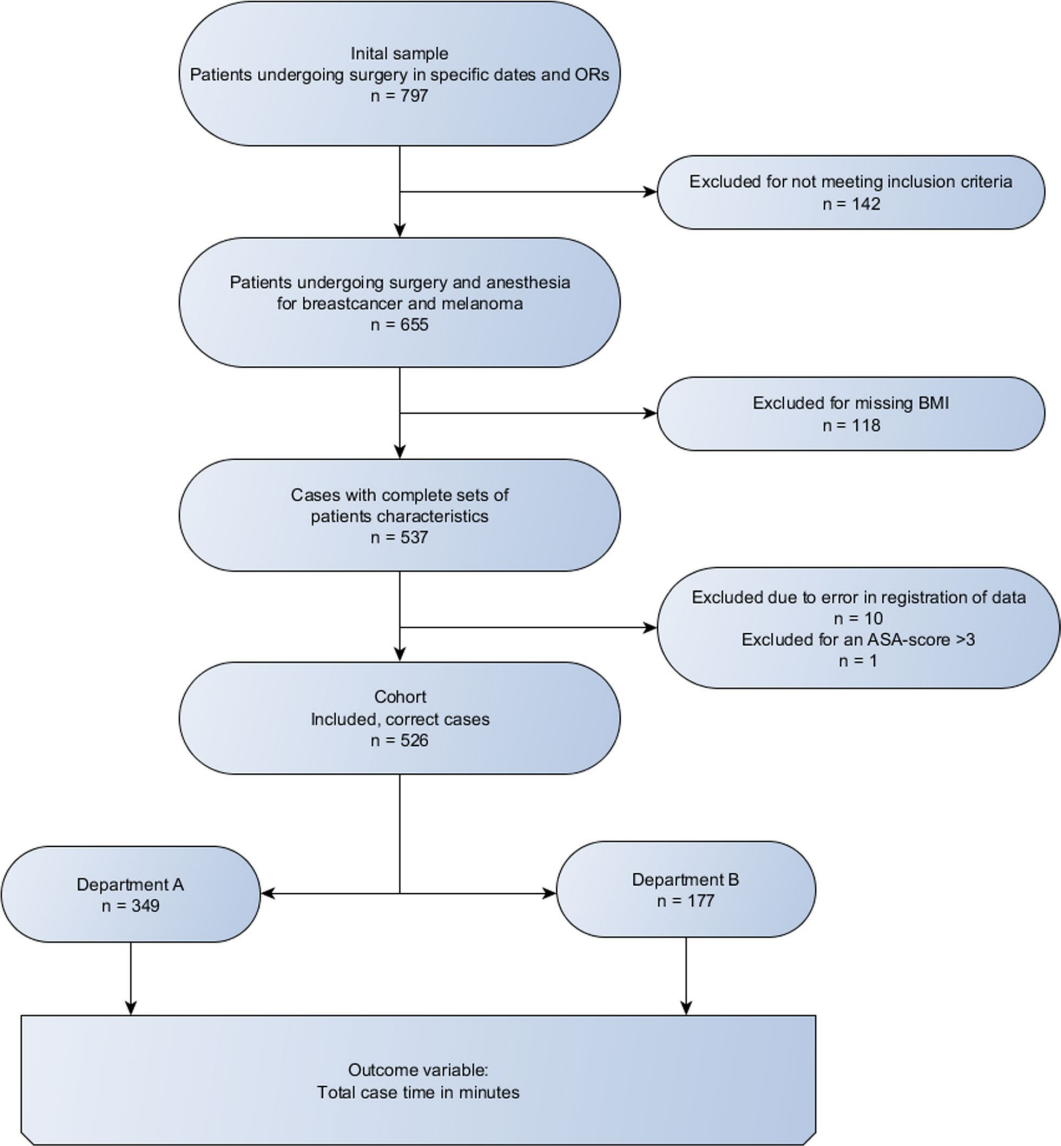
Flowchart of the inclusion process. From a total population of 797 eligible patients, a final cohort of 526 patients was included in the analysis

A total of 797 eligible patients were identified in the chosen time frame, but we excluded 142 of these patients due to not having one of the pre-specified diagnoses. Another 118 patients were excluded because of missing patient data (i.e., BMI). Ten more patients were excluded due to time registration errors in the Orbit program, where the most frequent error was two or more surgeries being registered as taking place in the same room at the same time. Finally, one patient was excluded because they were the only individual with an ASA score of 4. After these exclusions, the final study cohort comprised 526 patients (Fig. 1).

As mentioned previously, several covariates, including clinical, staffing, and patient characteristics, were available and used in the analysis. We summarized these covariates in Table 4. The differences between patients allocated to the two departments were small overall, except for the distributions of ASA scores and procedure groups.

**Table 4:** Characteristics of patients and operations

Characteristic	Total cohort n = 526	GS Department n = 349	CT Department n = 177
Age, years	60.27 (15.43)	60.98 (15.15)	58.88 (15.91)
BMI	26.40 (4.79)	26.57 (4.84)	26.06 (4.68)
ASA score 1 (%)	27.4	25.8	30.4
ASA score 2 (%)	59.9	58.7	62.3
ASA score 3 (%)	12.7	15.6	7.3
Sex—male (%)	12.0	13.8	9.0
Procedure group 1 (Extensive BC) (%)	16.9	18.1	14.7
Procedure group 2 (Limited BC) (%)	64.8	58.5	77.4
Procedure group 3 (Melanoma) (%)	18.3	23.5	7.9
Consultant present (%)	80.2	78.8	83.1
High-volume surgeon present (%)	85.0	83.7	87.6

Values are given as mean (standard deviation) or percentage of the cohort

*GS* general surgery, *CT* cardiothoracic surgery, *ASA* American Society of Anesthesiologists, *BC* breast cancer, *BMI* body mass index

### Difference in total case time

The average unadjusted difference in total case time between the two departments was 40.39 min: 206.96 min (standard deviation 60.89) at the GS department and 166.57 min (49.35) at the CT department (p < 0.001). Table 5 gives a breakdown of the three main phases of the operations defined by the PTG terminology, and their unadjusted time consumption. Notably, in this setting, the first four steps outlined by the PTG occur in parallel with the clinical preparations (e.g., RSS) and are not accounted for in time measurement and not included in the table. They are also outside the scope of this study because the focus is on the perioperative part, and all the surrounding aspects are not affected by which department will perform the procedure.

**Table 5:** Unadjusted mean time consumption of the three main phases of the surgical procedure

Phase (as defined by PTG)	Total cohort n = 526	GS Department n = 349	CT Department n = 177
Room set-up start—Preparation complete	64.92	71.30	52.10
Procedure physician of record in—Procedure-surgery finish	93.35	97.79	84.59
Patient out of room—Room clean-up finished	35.24	38.06	29.87

Values are in minutes

*PTG* Procedural Time Glossary, *GS* general surgery, *CT* cardiothoracic surgery

Adjusting for covariates in a linear regression model, the estimated regression coefficients are shown in Table 6 with their corresponding standard errors and p-values. After adjusting for the selected covariates, the difference in total case time remained significant, with patients who underwent surgical procedures at the CT department having a 30.67-min shorter total case time on average than those at the GS department (p < 0.001, 95% confidence interval − 40.02 to − 21.32). Differences in average operation time are estimated in our model because dummy variables for procedure groups were included, and we investigated a possible interaction effect between procedure group and operating department. However, a separate subgroup analysis would allow for coefficients of the control variables (such as age or sex) to differ between procedure groups, which our model did not. The estimated effect of interest was similar in all three procedure groups (data not shown), though other coefficients changed in both magnitude and sign.

**Table 6:** Variables with their corresponding estimated coefficients, standard errors, and p-values. R^2^ = 0.31

Variable	Estimate	Std. error	p-value
Intercept	151.08	19.55	0.000
CT department	− 30.67	4.75	0.000
Age	0.28	0.16	0.08
BMI	1.38	0.49	0.005
Sex—male	7.32	8.12	0.37
ASA score 2	14.73	5.61	0.009
ASA score 3	26.70	8.66	0.002
Procedure group 2 (Limited BC)	− 49.07	6.06	0.000
Procedure group 3 (Melanoma)	− 23.38	8.38	0.008
Presence of surgical trainee	19.99	5.72	0.000
Consultant present	− 2.40	6.64	0.72
High-volume surgeon present	0.36	8.17	0.96

*GS* general surgery, *CT* cardiothoracic surgery, *ASA* American Society of Anesthesiologists, *BC* breast cancer, *BMI* body mass index

### Missingness in the patient characteristics

Only the BMI variable had missing data, but the results were similar when mean-imputation of BMI was performed and an indicator for missingness added to the regression model (estimate = − 31.94, p < 0.001), with a 95% confidence interval of − 40.81 to − 23.07 for the estimated effect of department assignment on total case time. In the two sensitivity analyses for the missing completely at random assumption, the results were similar to the results obtained through listwise deletion, with the 95% confidence intervals for the effect of department allocation being − 38.10 to − 21.01 (estimate = − 29.55, p < 0.001) and − 41.65 to − 24.69 (estimate = − 33.17, p < 0.001) for the two imputation schemes, respectively.

## Discussion

The aim of this study was to investigate how the average total case time differs between two differently organized surgical departments at which the same surgeons operated on similar patients using largely standardized procedures for breast cancer and malignant melanoma. The estimated effect of being assigned to the CT department rather than the GS department on total case time was 30.67 min (p < 0.001).

A possible reason for the difference may be that the CT department has a higher overall level of education among its nursing staff, theoretically enabling a higher grade of flexibility within the team. Several studies have suggested that using the same team in the OR can have a large effect on efficiency [26–30]. Though none of the two departments use fixed teams for their operations, the CT department has fewer staff, which may lead to similar effects as observed in the above studies. This could mean that the nursing staff becomes more accustomed to working together, reducing distractions and delays [31]. As for the skill mix and level of theoretical knowledge within the OR team, specifically the presence of more than one ORN on the team, the GS department never staffed two ORNs, whereas the CT department had two ORNs in 77% of cases. Earlier research found a critical link between technical skills, theoretical knowledge, and overall competence within the OR team [32–34].

Another argument regarding the observed differences is that larger departments need a more “flexible” staffing policy because they must be prepared for a greater number of emergencies. However, the CT department is smaller (regarding total caseload) and it still needs to keep resources available for acute cardiothoracic procedures. Therefore, we argue that it should be a priority for management to accommodate the overall flexibility. One framework for this is the acute care surgery model described by Wanis et al. [35], who found a decrease in wait time until surgery and the proportion of after-hours emergency surgeries, without the threat of displacing less acute and elective surgery.

Notably there are more teaching activities at the GS department than the CT department in terms of both nurses getting their specialization degrees and pre-graduation doctors, though the inclusion of the student covariates in our regression model did not change the results in any meaningful way (data not shown). Given that there was some uncertainty regarding whether the presence of these students was determined before or after the patient was allocated to a department (i.e., if it was part of the effect we wished to estimate), we opted not to include it in our regression model. In regards to post-graduated doctors, such as anesthesia and surgical trainees, there were no discernable differences in attendance in the OR. Previous studies have found that, when anesthesia trainees get hands-on education in the OR, it seldom increased the total case time to a high degree [36, 37]. The presence, or perhaps the active participation, of surgical trainees has been shown to increase the operating time in some mastectomy cases, though it became less apparent with experience [38]. As the experience of the participating surgical trainees is hard to measure objectively, adjusting for it is difficult. To mitigate this, however, we created two covariates and adjusted for them accordingly: ‘consultant present’, which refers to the formal competence of the surgeon (i.e., if they are a consultant or not), and ‘high-volume surgeon present’, which relates to the amount of surgery performed by a surgeon.

### Generalizability

There are limitations in comparing the Swedish healthcare system to, for example, the US healthcare system. With a population of almost 360 million versus Sweden’s 10 million, the US spent $10,624 per capita, or 17% of its gross domestic production (GDP), on healthcare in 2018, whereas Sweden spent $5982 per capita, or 11% of its GDP [39, 40]. In regards to hospital ranking, the fact that 4 of the top 10 hospitals in the world are in the US [41] says a lot about the competence, quality, and potential of the US healthcare sector. Sweden, however, also ranks high with one hospital in 7th place [41]. In a comparison of healthcare performance by the Commonwealth Fund, the US was ranked 11th and Sweden 7th [42]. Although the US system heavily outranks most others when it comes to the metrics of care processes, they are ranked last on all others [42], showing that discussions about socialized medicine are complex and multifaceted. This leads us to believe that the present findings may be one small piece of the puzzle in promoting a more cost-efficient way of delivering healthcare in terms of OR department organization.

## Limitations

There are a few potential limitations to this study. Even though there is potential for selection bias when analyzing cases from only certain months in the GS department, we have no reason to suspect that the total case time should vary with the calendar month. Another potential source of bias is the time registrations performed by the OR team. However, due to the temporal proximity to the observed events, the effect is likely marginal. Inaccuracy in time registration was found in 10 cases, where the person responsible for the registration was not following routines. These cases were excluded during the inclusion phase and we observed no further discernable pattern of the error. Lastly, the overrepresentation of malignant melanoma cases at the GS department after 2013 may pose a source of bias. That said, interaction terms between procedure group and allocated department did not show any significance and, if included, did not affect the results in any substantial way.

## Conclusion

After adjusting for select clinically relevant covariates, patients who underwent surgical procedures at the CT department had a 30.67-min shorter total case time on average than those at the GS department (p < 0.001, 95% confidence interval − 40.02 to − 21.32). The results of the present study could be used as a foundation for discussions on how to organize a more productive surgical department. As the departmental differences are at an organizational level, we think the results may also be generalized to other surgical contexts and departments.

A health-economic evaluation including the two departments would be of great interest in future studies and may further the arguments for developing OR department organization.

## Supplementary Information

**Additional file 1: Figure S1. **Patient diagnosis and departmental selection process. The process for the patients from suspicion through diagnosis and up to time of surgery.

## Data Availability

Data are not publicly available. For inquiries, contact the corresponding author.

## Abbreviations

GS: General surgery
CT: Cardiothoracic
ASA: American Society of Anesthesiologists
BMI: Body mass index
OR: Operating room
ORN: Operating room nurse
CCM: Classification of care measures
BC: Breast cancer

## Declarations

### Methodological declaration

All our data collection and analytical methods were carried out in accordance with relevant guidelines and regulations.
