# Supplementary material for: Productivity in relation to organization of a surgical department: a retrospective observational study
Source: BMC Surg. 2022 Mar 24;22:114. doi: 10.1186/s12893-022-01563-6 (PMC8953785; doi:10.1186/s12893-022-01563-6)
Supplement: Supplementary file 1 — Additional file 1: Figure S1. Patient diagnosis and departmental selection process. The process for the patients from suspicion through diagnosis and up to time of surgery. [file 12893_2022_1563_MOESM1_ESM.docx]

**Additional file 1 – Patient diagnosis and departmental selection process**


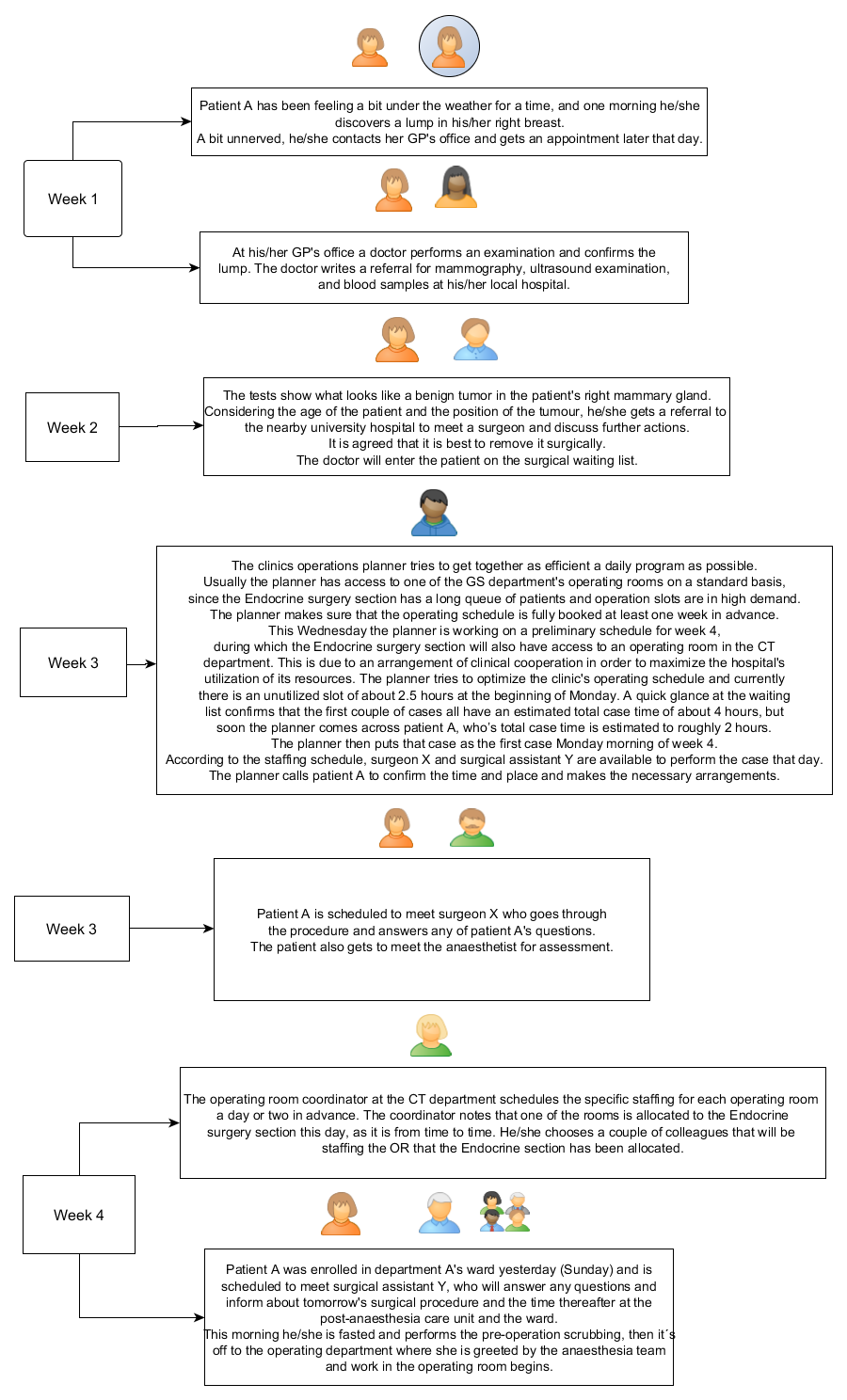


*Figure S1 – Patient diagnosis and departmental selection process. The process for the patients from suspicion through diagnosis and up to time of surgery.*
